# Revealing protein structures: crystallization of protein‐ligand complexes – co‐crystallization and crystal soaking

**DOI:** 10.1002/2211-5463.13913

**Published:** 2024-10-20

**Authors:** Barbora Kaščáková, Anna Koutská, Michaela Burdová, Petra Havlíčková, Ivana Kutá Smatanová

**Affiliations:** ^1^ Department of Chemistry, Faculty of Science University of South Bohemia in České Budějovice Czech Republic; ^2^ Department of Molecular Biology and Genetics, Faculty of Science University of South Bohemia in České Budějovice Czech Republic

**Keywords:** advanced crystallization, co‐crystallization, crystal soaking, crystallization protocol, microseeding

## Abstract

Protein crystallogenesis represents a key step in X‐ray crystallography studies that employ co‐crystallization and ligand soaking for investigating ligand binding to proteins. Co‐crystallization is a method that enables the precise determination of binding positions, although it necessitates a significant degree of optimization. The utilization of microseeding can facilitate a reduction in sample requirements and accelerate the co‐crystallization process. Ligand soaking is the preferred method due to its simplicity; however, it requires careful control of soaking conditions to ensure the successful integration of the ligands. This research protocol details the procedures for co‐crystallization and soaking to achieve protein–ligand complex formation, which is essential for advancing drug discovery. Additionally, a simple protocol for demonstrating soaking for educational purposes is described.

## Graphical abstract

Co‐crystallization crystallizes a protein with its ligand, resulting in protein–ligand complex crystals. In contrast, soaking introduces a ligand into preformed protein crystals, allowing it to bind. Both methods produce crystals for X‐ray diffraction, which generates diffraction patterns that are analyzed to determine the three‐dimensional structure of the complex. This process uncovers key interactions critical to understanding the protein's biological functions.

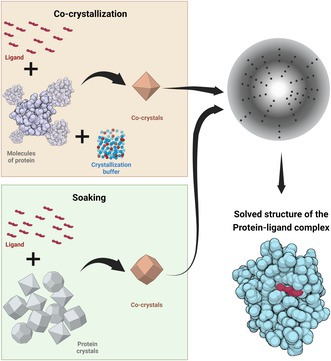

**Abbreviations**
DLS: dynamic light scattering
DMSO: dimethyl sulfoxide
DSF: differential scanning fluorimetry
DTT: dithiothreitol
EDTA: ethylenediaminetetraacetic acid
FBDD: fragment‐based drug design
His6: hexa histidine‐tag
IPTG: isopropyl β‐d‐1‐thiogalactopyranoside
ITC: isothermal titration calorimetry
*K*_d_
: equilibrium dissociation constant
*K*_M_
: Michaelis constant
MALS: multi‐angle light scattering
MWCO: molecular weight cutoff
PEG: polyethylene glycol
RI: refractive index
rMMS: random microseed matrix screening
SADD: structure‐aided drug design
SEC: size exclusion chromatography
SPR: surface plasmon resonance

X‐ray crystallography is a widely used method for elucidating the binding of ligands (small molecules, nucleic acids, organic, or inorganic compounds—substrates, cofactors, peptides, or ions) to a target protein [1, 2]. Structure‐aided drug design (SADD) employs crystallography to design new drugs based on atomic‐resolution data from already‐known protein–ligand complex structures, potentially resulting in highly specific and potent drugs [3]. In contrast, fragment‐based drug design (FBDD) commences with simple fragments and progresses to larger molecules, focusing on binding interactions rather than comprehensive structural optimization, potentially facilitating the discovery of novel scaffolds while constraining compound optimization [4]. Various methods have been developed to obtain protein–ligand crystals, including protein engineering, co‐expression, co‐purification, co‐crystallization, and ligand soaking [3, 5, 6] (Fig. 1).

**Fig. 1:**
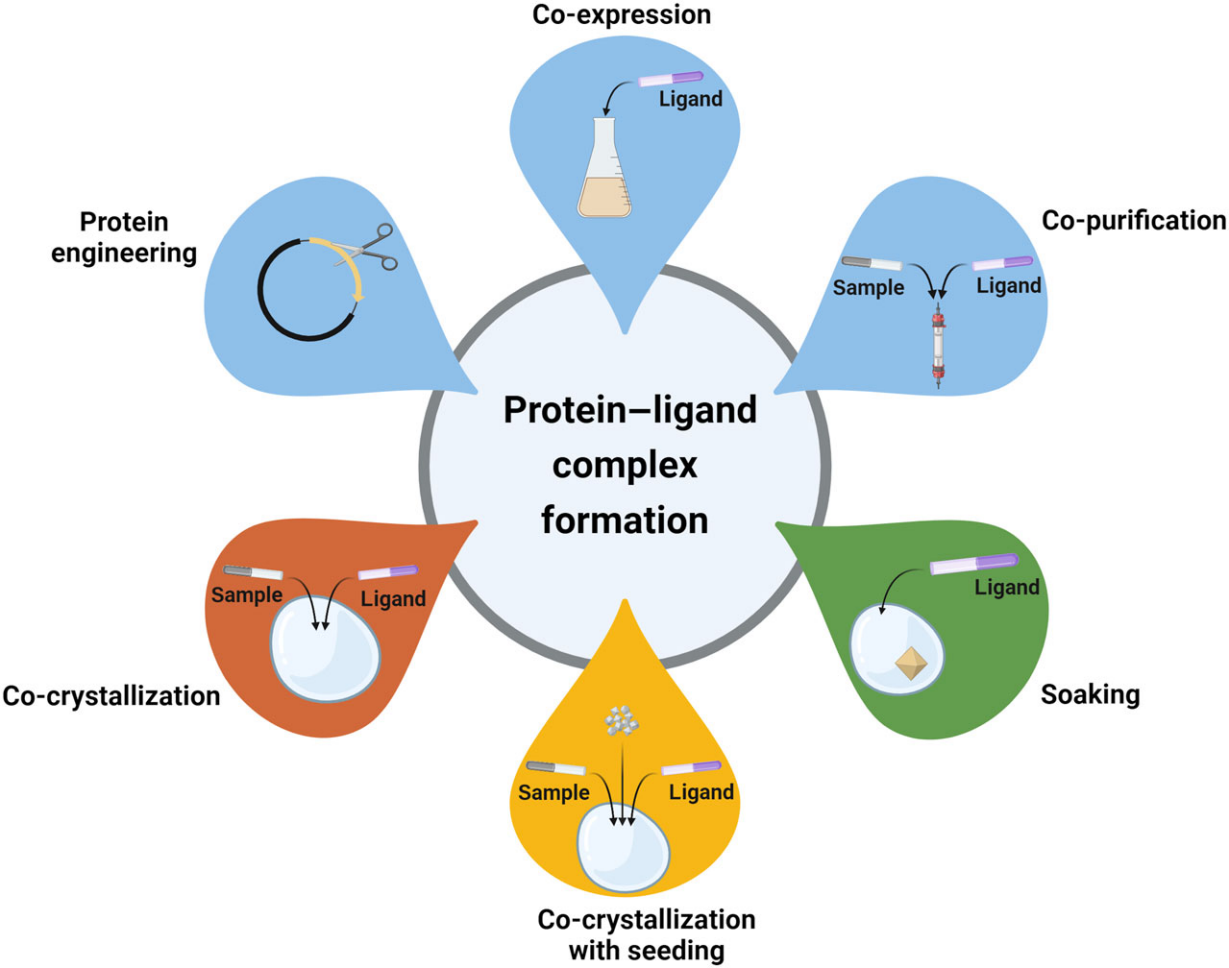
Methodologies employed in the investigation of protein–ligand complex formation. Depicted techniques include protein engineering, co‐expression, co‐purification, and co‐crystallization, as well as advanced approaches such as co‐crystallization with microseeding and soaking. The design of the construct is a pivotal preliminary stage in the process of crystallization, particularly in the context of studying protein–ligand interactions. Protein engineering can be employed for the purpose of isolating the region of protein–ligand interaction, removing disordered regions, or adding affinity tags to enhance protein solubility (for further details, see Müller [6]). The process of optimizing sample preparation requires the selection of an appropriate recombinant protein expression system [5, 7]. The expression of proteins with ligands has the potential to markedly enhance protein expression levels and solubility, depending on several factors, including the potency, solubility, and availability of the ligand, along with the DMSO concentration. The introduction of mutations can also serve to enhance protein expression in the presence of ligands [5]. Should the protein quality remain inadequate, incorporating ligands during purification can enhance stability and solubility while reducing aggregation. The utilization of ligands during cell lysis, purification, or refolding enables the displacement of other proteins, such as HSP90, thereby improving the overall protein quality [5]. Furthermore, ligands may occasionally function as a crystallization agent for proteins that would otherwise be non‐crystallizable [5, 6, 7]. Created with BioRender.com.

Co‐crystallization entails exposing the protein to the ligand in solution at the beginning of the experiment [1, 3] (Fig. 2A). In comparison with soaking, co‐crystallization is more accurate for determining the correct ligand‐binding position [1], as crystal packing tends to favor the ligand position bound to the active site. Consequently, the space group of soaked crystals and those of ‘co‐crystals’ with the same ligand bound can be distinct. However, co‐crystallization is a more time‐consuming and costly process, necessitating the optimization of the protocol for each ligand due to the varying chemical properties of the ligands. This involves utilizing a range of solubilizers (organic solvents—DMSO, surfactants, cyclodextrins, etc.), additives (EDTA, DTT, different salts, etc.), or even varying crystallization conditions to obtain a ‘co‐crystal’ for different ligands [7]. It is essential to establish the ligand affinity and its concentration to ensure the utilization of a 10–1000‐fold excess over its *K*_d_ [8].

**Fig. 2:**
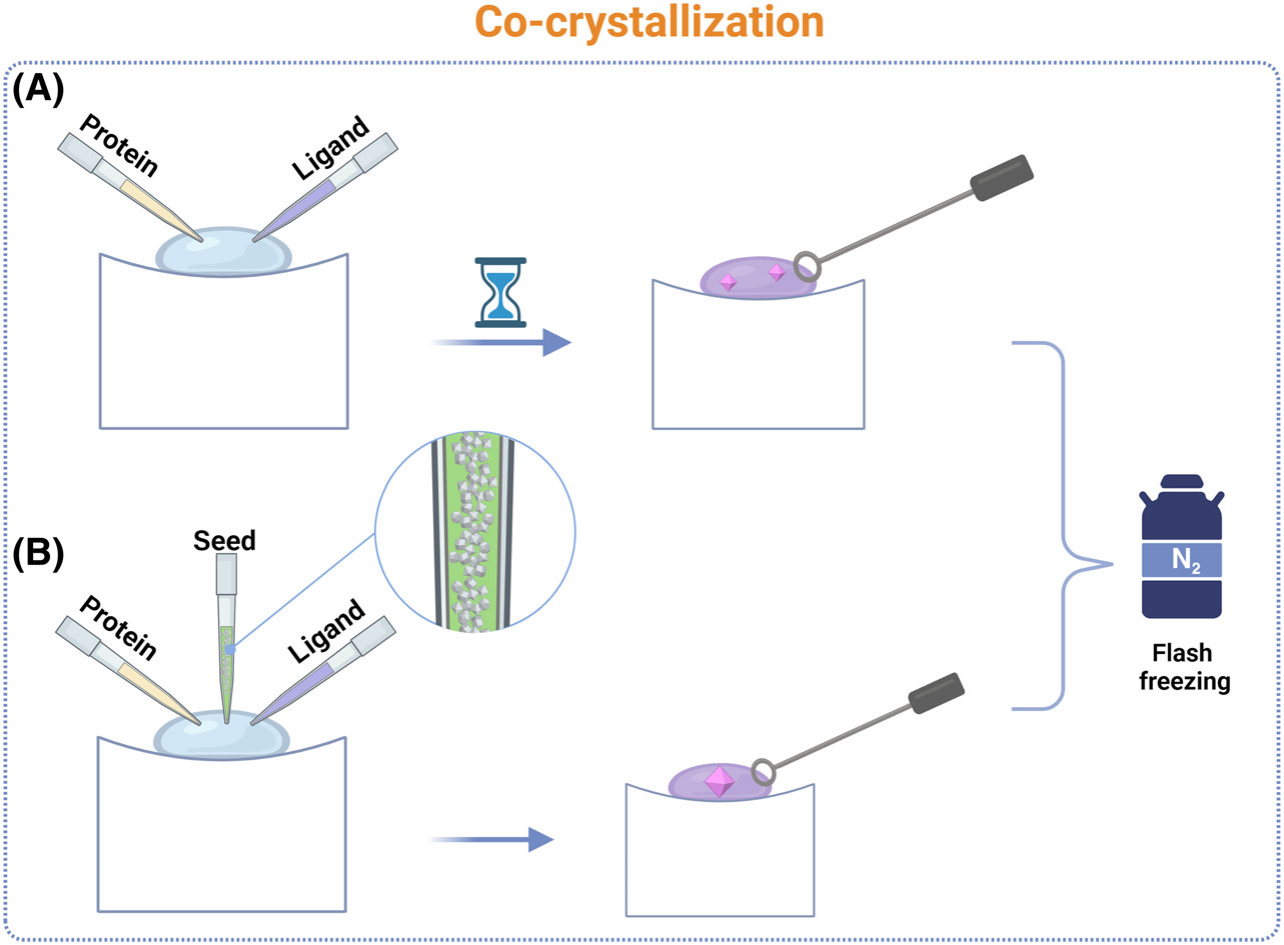
Schematic representation of co‐crystallization experiments. (A) The purified protein and ligand are mixed in a defined molar ratio to form a stable complex. (B) In the case of using microseeding, a small amount of seed stock is added to the crystallization drops or directly to the crystallization drop in order to promote crystal growth. These mixtures are then incubated for a specific period of time to ensure that the binding process is complete. A variety of crystallization conditions (including pH, precipitants, and additives) are screened using methods such as vapor diffusion (as illustrated by the example of sitting drop vapor diffusion). The initial crystals that form are then optimized by fine‐tuning the crystallization conditions to improve both their size and diffraction quality. Monocrystals may be cryoprotected prior to flash‐cooling in liquid nitrogen. Created with BioRender.com.

Co‐crystallization utilizing microseeding alongside automated crystallization accelerates the process while reducing the need for sample [6] (Fig. 2B). Microseeding bypasses the nucleation zone, proceeding directly to the crystal growth in the metastable zone, which accelerates crystallization. Microcrystals, microcrystalline precipitate, dendric crystals, or spherulites can be employed as microseeds to facilitate crystallization even if the protein does not form monocrystals [9, 10].

Ligand soaking is preferred due to its simplicity, whereby the ligand solution is applied directly to already‐formed well‐diffracting protein crystals (Fig. 3). Nevertheless, the crystallization conditions must be optimized [1, 3]. The solvent channels in protein crystals, a consequence of crystal packing, are primarily filled with crystallization buffer (up to 80% solvent), allowing the ligand's free diffusion into its functional binding site. The rate of diffusion is influenced by the ligand concentration and its affinity [3, 11]. The process may take between a few seconds (small to nanocrystals) [12] and several days (in case of replacement soaking method [13]) for the ligand to fully populate in the binding site. It is essential to consider the diffusion kinetics during the soaking process, as ensuring accessibility of the binding site for ligands is crucial for proteins in crystalline form [6]. Additionally, factors such as crystal fragility and its frequent sensitivity to solvents may require the use of stabilization buffers, ligand cross‐linking to the protein, and precise control of soaking time and ligand concentration. Additives can enhance ligand binding during soaking and cryoprotectant exchange [5, 8]. Large conformational changes during soaking could result in the cracking or dissolution of the crystal. Sometimes, however, the rearrangement of the crystal lattice improves crystallinity, leading to the formation of stable complex crystals (Fig. 3) [3, 7, 14]. Moreover, exchanging ligands in preformed stable ‘co‐crystals’ through the replacement soaking method has been effective in reducing the necessity for multiple apo‐protein crystals [3, 5, 15].

**Fig. 3:**
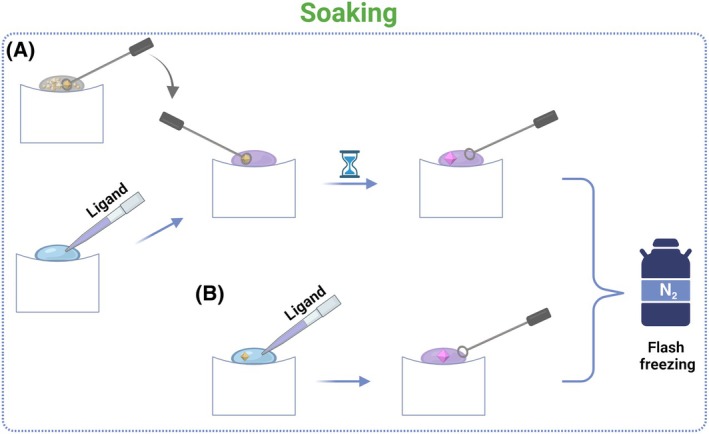
Schematic representation of the ligand soaking into crystals. The soaking solution, containing the ligand at a desired concentration, is prepared. (A) The apo‐protein crystals are carefully transferred to the soaking solution using specialized tools (e.g., cryo‐loops and pipettes). (B) Alternatively, the concentrated ligand can be added directly to the crystallization drop with the crystal/s. In this step, it is possible to enrich the soaking solution with a cryoprotectant solution. Subsequently, the crystals are incubated in the soaking solution for a defined time. During this period, the ligand diffuses into the crystals and binds to the protein. The crystals are then flash‐cooled in liquid nitrogen. Created with BioRender.com.

This research protocol describes common experiments for obtaining protein crystals with bound ligands by co‐crystallization and soaking using the vapor diffusion method:

1. Co‐crystallization protocol for protein–ligand complex formation (with optional use of seeding).
2. Soaking protocol for protein–ligand complex formation.
3. Soaking protocol for educational purposes using a model protein.

## Materials

### Purification of the macromolecules for complex crystallization

1. Protein Galectin‐9C^His6^ from Homo sapiens (monomer of 156 amino acids, 17.7 kDa) and its substrate (small‐sized carbohydrate with MM of 762.7 g·mol^−1^) used for further crystallization were provided by kind support due to cooperation with the Laboratory of Biotransformation, Institute of Microbiology of the Czech Academy of Sciences.
2. Superdex™ 75 Increase 10/300 GL for size exclusion chromatography (#29148721; Cytiva, Karlsruhe, Germany).
3. MALS detector (DAWN Heleos II; Wyatt Technologies, Dernbach, Germany) and an RI detector (Optilab T‐rEX, Wyatt).

### Stabilization of the macromolecules for further co‐crystallization

1. Buffer exchange and protein concentration.
  - Amicon® Ultra‐15 Centrifugal Filter Unit with 10 kDa MWCO (#UFC9010; Merck Millipore, Germany).
  - Amicon® Ultra Centrifugal Filter, 3 kDa MWCO (#UFC500308; Merck Millipore, Praha, Czech Republic).
2. Dilution of used proteins and ligands.
  - Deionized water or 50 mm sodium acetate buffer of pH 4.6 for Lysozyme.
  - 50 mm sodium phosphate pH 8.0, 100 mm NaCl for Galectin.
  - DMSO.
  - Vortex mixer.

### Ultracentrifugation

1. High‐Speed Refrigerated Microcentrifuge set on 20 000 *g* and 4 °C.
2. 1.5/2 mL centrifugation tubes.

### Crystallization

1. 96‐well sitting‐drop crystallization plates (MRC 2 Lens Crystallisation Plate; SWISCI, Buckinghamshire, UK).
2. 96‐well sitting‐drop crystallization plates (3 Lens Crystallisation Plate; SWISCI).
3. 24‐well sitting drop/hanging drop pregreased crystallization plates (MiTeGen, Jena, Germany).
4. Siliconized cover slides (Hampton Research, Aliso Viejo, CA, USA).
5. Sterile multichannel pipettes with sterile tips.
6. Pipettes with sterile tips for pipetting small volumes (0.1–10 μL).
7. Oryx 8 crystallization robot (Douglass Instruments, Hungerford, UK).
8. Commercially available or home‐made crystallization screens (e.g., SG1, Morpheus II, PACT premier; Molecular Dimensions, Rotherham, UK).
9. Crystal crusher (Hampton Research).
10. Seed Bead™ Kits (Hampton Research).
11. Micro‐Tools Set and Seeding Tool (Hampton Research).
12. Crystal clear sealing tape (Hampton Research).
13. Microplate Sealing Sheets (e.g., ThermalSeal RTS™ Sealing Films, #Z742256; Merc Millipore).
14. Tweezers.
15. Scalpel for opening the drops.
16. Incubators set on 4/20 °C.
17. Lyophilized Lysozyme from chicken egg white (#SAE0152; Sigma‐Aldrich, Praha, Czech Republic).
18. Lysozyme's crystallization solution (50 mm sodium acetate pH 4.6, 30% w/v Polyethylene glycol 6000, 0.25 m NaCl).
19. IZIT dye (Hampton Research) or JBS Rainbow (Jena Bioscience, Jena, Germany).

## Methods

### Purification of the macromolecules for crystallization

1. Purify the protein using size exclusion chromatography (SEC), optionally SEC‐MALS, to obtain a highly pure, homogenous, and monodisperse sample.

### Preparation of macromolecules for crystallization

1. Protein preparation:
  - Select a simple buffer with appropriate ionic strength (e.g., NaCl) and additives to maintain sample solubility, stability, activity, and homogeneity.
  - Ensure that the protein is at least 95% pure, as confirmed at least by Coomassie‐stained SDS/PAGE. Check protein stability, homogeneity, monodispersity, and activity by DLS, DSF, or other preferred method [10, 16].
  - Concentrate the protein to the maximum stable concentration (5–25 mg·mL^−1^) and, if necessary, filter through a 0.22 μm filter or centrifuge at 15 000 r.c.f. for 10–15 min at 4 °C to remove aggregates and precipitates [17, 18].
  - If necessary, use buffer exchange to remove excess salts (more than 150 mm) or stabilizers that could inhibit crystallization using Amicon centrifugation filters with appropriate MWCO [10].
2. Lysozyme preparation:
  - Weigh the desired amount of lysozyme powder and dissolve it by slowly adding the solvent of choice.
  - Mix the solution gently, for example, by vortexing.
  - The solution may be filtered through a 0.22 μm filter or centrifuged as stated above.
  - Store the lysozyme solution at 4 °C [18].
  - Lysozyme at a concentration of 60 mg·mL^−1^ or less should be used for this protocol.
3. Ligand preparation:
  - Dissolve the ligand in a suitable solvent (e.g., 100% DMSO [5]) to make a concentrated stock solution (e.g., 100 mm) [3].
  - Dilute the stock solution to the desired working concentration (e.g., 5 mm [15], or typically 10–1000‐fold the dissociation constant (*K*_d_) if known [5]) in a protein‐compatible buffer (e.g., a final DMSO concentration of 3–5%). If solubility problems occur, add the dry compound directly to the protein solution or onto the crystal for overnight incubation [5]. Alternatively, deposit a drop of ligand in DMSO into the crystallization wells, allow the DMSO to evaporate, forming a ligand film, and then prepare the crystallization drop as usual [19].

### Co‐crystallization protocol with optional seeding step

**Fig. 4:**
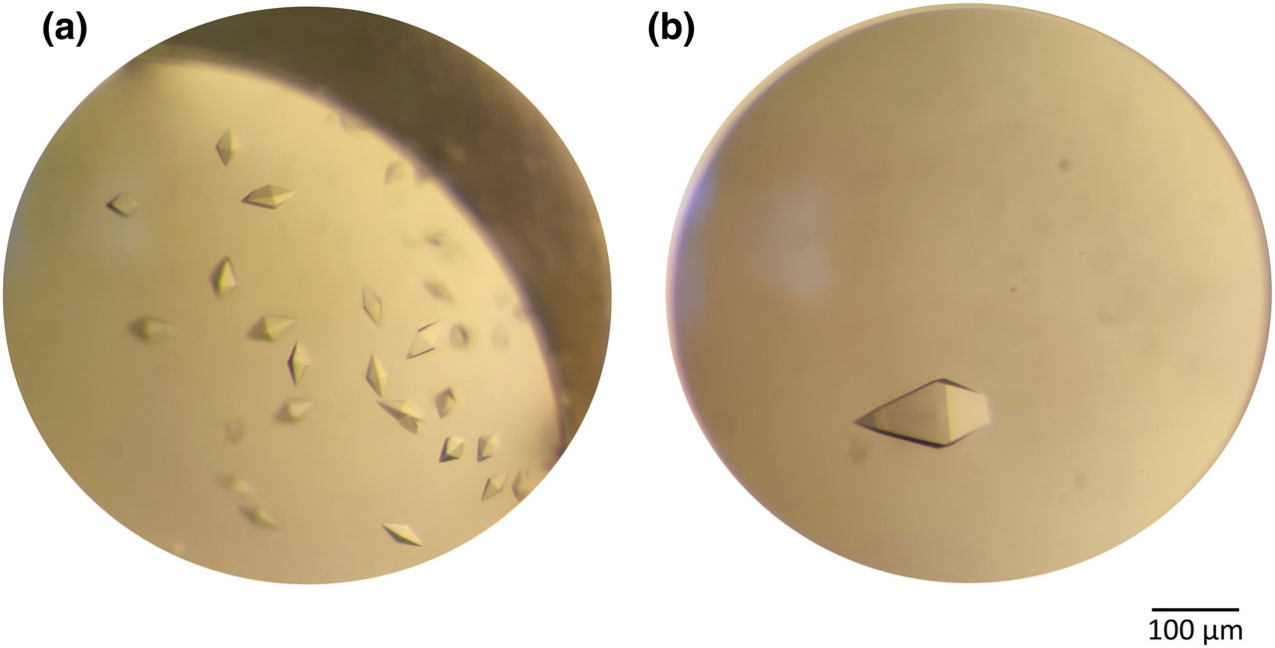
Visualization of the Galectin‐9C^His6^ crystals. (A) The protein crystals co‐crystallized with the selected ligand in a solution of 0.2 m Sodium sulfate, 20% w/v PEG 3350, and 10% v/v Ethylene glycol. The crystals grew over a period of 6 weeks. (B) The monocrystal was obtained in 0.1 m MES‐NaOH pH 6, 0.1 m Magnesium chloride hexahydrate, 20% w/v PEG 6000, and 10% v/v Ethylene glycol. Crystal was used for the apo‐protein soaking experiment. Both were visualized under a light microscope in a 1 μL drop with a 1 : 1 ratio.

1. Mixing of protein and ligand:
  - Mix the protein solution with the ligand solution in the appropriate molar ratio, often starting with equimolar concentrations.
  - Incubate the mixture at 4 °C for a sufficient time (e.g., 1–2 h or overnight) to allow the complex to form.
  - If precipitation occurs, spin the sample, and use the supernatant for crystallization [6].
2. Initial crystallization screening:
  - Use commercial crystallization screens (e.g., Hampton Research, Molecular Dimensions, and Jena Bioscience) to determine initial crystallization conditions. Stir thoroughly before using crystallization screens. Pipette 50–100 μL of precipitant into the crystallization plate reservoir.
  - Set up crystallization drops using the vapor diffusion method (hanging drop or sitting drop) by mixing equal volumes of protein–ligand complex and reservoir solution (typically 1 μL + 1 μL). In multi‐drop crystallization plates, use different ratios of protein–ligand complex and reservoir solution to ensure a wider range of screening [20].
  - Seal the droplets and incubate at 4 °C or 20 °C (or any preferred temperature) to promote crystallization (Fig. 4).
3. Optional step—microseeding:
  - Prepare seed stock by finely crushing small amounts of previously grown microcrystals or small monocrystals in the mother liquor with a seed bead in a centrifugation tube and mixing with vortex or with crystal crusher in the drop well (follow manufacturer's manual: HR2‐320 Seed Bead kit User Guide, HR4‐216 Crystal Crusher User Guide). Transfer seeds into a stabilizing solution in a centrifugation tube.
  - Perform serial dilutions of the seed stock to obtain a range of seed concentrations (follow HR2‐320 Seed Bead Kit User Guide). Typically, a 100‐fold dilution is sufficient, depending on the number and size of crushed crystals (alternatively 10^2^–10^7^ dilution).
  - Inject a small volume (0.1–0.5 μL, depending on drop final volume, for example, 0.1–1 μL drop) of the selected seed stock dilution into fresh crystallization drops containing the protein–ligand mixture [21]. Optionally, a small amount of concentrated seed stock can be added directly to the protein–ligand mixture (e.g., 10 μL into 1 mL) right before setting up the crystallization plate. Alternatively, streak‐seeding can be used [9].
  - Seal the drops and incubate at 4 °C or 20 °C to promote crystallization.

### Crystal soaking protocol

1. Preparation of protein crystals:
  - Grow protein crystals using vapor diffusion or another suitable method (Fig. 4).
  - Ensure that the crystals are of high diffraction quality (resolution better than 2.5 Å) [4].
2. Soaking procedure:
  - Transfer the ligand into a droplet with the protein crystal by carefully pipetting the ligand‐working solution (the amount depends on the drop size, e.g., for a 2 μL drop, pipette 0.1–0.2 μL of concentrated ligand) into the crystallization drop where the protein crystals are growing without disturbing the crystals [6].
  - Alternatively, the protein crystal(s) can be carefully transferred into the ligand‐soaking solution using a cryo‐loop or pipette.
  - Incubate the crystals in the ligand solution for typically 10–30 min, or longer, if necessary (up to several days [5, 15]), to ensure adequate binding to the accessible binding site. To determine the optimal soaking time, add the ligand to the crystallization droplets, harvest, and flash‐cool crystals at different intervals. The ideal soaking time can then be identified by comparing Fo‐Fc maps of the docked ligand.

### Harvesting and flash‐cooling of crystals

1. Harvesting:
  - Position the plate under a stereo microscope and focus on the well that contains crystals.
  - Open the drop and add a small volume of precipitant solution into the crystallization drop (0.5–1 μL to avoid dissolving the crystals) to prevent dehydration. It is important to note that the crystallization solution should be free of cryoprotectants, as any manipulation of the crystals may impact their quality and suitability for further data collection [22].
2. Cryoprotection:
  - For cryoprotection, cryoprotectant solution (e.g., 20–30% glycerol, ethylene glycol, or glucose in soaking solution) should be added to the soaking solution or droplet to protect the crystals during its flash‐cooling [22]. Alternatively, the crystal can be harvested with a cryo‐loop and immersed in a drop of cryoprotectant solution prepared on parafilm or coverslip.
  - Incubate the crystal for a period of between 5 s and 5 min before flash‐cooling. It is advisable to examine the crystals for any signs of cracking or dissolution [22].
  - If convenient, ligands can be added to the cryoprotectant solution, facilitating simultaneous ligand soaking and cryoprotection. This method is particularly beneficial for crystals that are highly sensitive to rapid changes in their environment, thus ensuring higher success rates in preserving crystal integrity during flash‐cooling [6].
  - Ensure that the final cryoprotectant concentration is compatible with both the crystal and the ligand.
3. Flash‐cooling:
  - Carefully mount the crystals in cryo‐loops and flash‐cooled them in liquid nitrogen.
  - Transfer the crystals to a cryogenic dewar and ship them to the synchrotron facilities for data collection.

### Crystal soaking experiment for teaching purposes

Commercially available crystal dyes can be used to demonstrate the ligand‐soaking experiment [23]. The use of the small molecule dye for lysozyme (or any model protein) crystal soaking is a creative way to explain the process of incorporation of ligands into the intermolecular solvent channels of the crystal. It can demonstrate the rate of the ligand soaking into the crystal as well as confirm the biomolecular origin of the crystals formed.

**Fig. 5:**
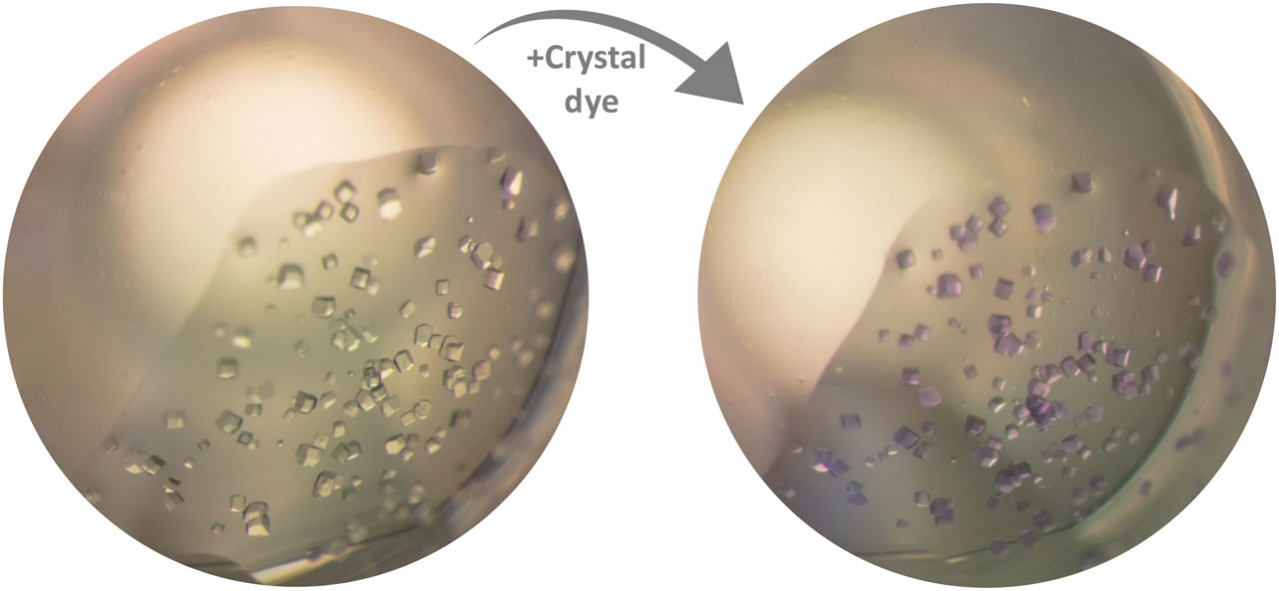
Visualization of the soaking experiment. The lysozyme crystals before staining (left) were grown under the following conditions: 60 mg·mL^−1^ of lysozyme in 50 mm sodium acetate pH 4.6, 30% w/v PEG 6000 and 0.25 m NaCl (2 μL : 2 μL) after 30 min at 20 °C were visualized under the light microscope. The same crystallization droplet after the application of a crystallization dye (right) and an incubation of 30 min. The dye enhances the contrast and makes the crystals appear prominently colored.

1. Pipette 200 μL lysozyme's crystallization solution into the wells of a 24‐well drop plate.
2. Pipette equal volumes of protein sample (60 mg·mL^−1^) and reservoir solution (2 μL + 2 μL) into the wells of the plate. Adjust the volumes as necessary. Using more protein than precipitant can help to control crystal growth. Avoid bubble formation when pipetting solutions.
3. Cover the wells with optically clear sealing tape. Incubate at 20 °C for more rapid crystal growth.
4. When sufficiently large monocrystals appear, open the drop and pipette 0.1–0.5 μL of diluted dye for IZIT Dye (diluted 1 : 1 with reservoir solution) or undiluted dye for JBS Rainbow, depending on the dyeing strength.
5. Reseal the well and incubate for 10–30 min until the dye is incorporated into the crystals.
6. Monitor the drop throughout the experiment (Fig. 5).

## Tips & tricks

1. Perform crystallization experiments even if protein purity is below 95%, as crystallization alone can be used for purification [16, 24].
2. Do not mix different purification batches [10].
3. Monitor drops regularly under a light microscope (×20–×100 magnification) to observe crystal growth.
4. Optimize conditions by adjusting precipitant concentration, buffer pH, temperature (e.g., 4 °C and 20 °C), additives (e.g., detergents, salts), and droplet ratios. If necessary, test changes in protein and ligand concentration ratios [25].
5. Determine the ligand‐binding affinity (e.g., SPR, ITC) for successful co‐crystallization. High affinity (nm to low mm range) is required, using a 5‐fold access of *K*_M_. Insufficient affinity of the heterogeneous solution (complexed and uncomplexed molecules) can hinder crystal formation [8].
6. When microseeding, determine the lowest precipitant concentration that will prevent microcrystals from melting and optimize the seed dilution to obtain large monocrystals. If many small microcrystals are present, further dilute the seed [10].
7. To avoid twinning in co‐crystals when using microseeds, which accelerates crystallization and affects the quality of the measured data [9], use a lower temperature (e.g., 4 °C) or the microbatch method to slow crystal growth and improve the quality.
8. Carefully optimize ligand‐soaking incubation time and concentrations of ligand and solubilizing solvent (e.g., DMSO). Balance protein and ligand stability, as crystals are often sensitive to the ligand solvent used, to achieve a reproducible soaking protocol [8].
9. If crystal packing obscures the binding site or crystals don't tolerate prolonged soaking or cryoprotectant solution, slowly transfer the crystal from 5% to 30% concentrated cryoprotectant/ligand or co‐crystallize [26].
10. If apo‐crystals are formed in PEGs or alcohols, try to dissolve the less soluble ligand in the mixture of DMSO and PEG/alcohol to achieve its solubility more easily [6].
11. If the crystal is soaked in the dry ligand, wash the crystal before flash‐cooling to remove traces of ligand precipitation that can cause weak diffraction [6].
12. To prevent crystal cracking during soaking, add 0.2–0.3% m/v low gelling point agarose to the crystallization drops [27]. The agarose matrix provides mechanical protection and creates a diffusive environment that facilitates smooth ligand penetration. Additionally, it may help prevent twinning and promote 3D crystal growth [28].

## Conflict of interest

The authors declare no conflict of interest.

### Peer review

The peer review history for this article is available at https://www.webofscience.com/api/gateway/wos/peer‐review/10.1002/2211‐5463.13913.

## Author contributions

BK and MB performed the SEC purification. BK, MB, and PH performed all crystallization experiments. BK and AK created the figures. BK wrote the research protocol. IKS contributed to the funding.

## Data Availability

The crystallization data that support the findings of this study are available from the first (karafb00@prf.jcu.cz) or corresponding author (kuta@prf.jcu.cz) upon reasonable request.
